# Elucidating the spatially varying relation between cervical cancer and socio-economic conditions in England

**DOI:** 10.1186/1476-072X-10-51

**Published:** 2011-09-26

**Authors:** Edith MY Cheng, Peter M Atkinson, Arjan K Shahani

**Affiliations:** 1 Centre for Geographical Health Research, Geography and Environment, University of Southampton, Highfield, Southampton, UK; 2 Faculty of Medicine, University of Southampton, Highfield, Southampton, UK

**Keywords:** Geographically weighted regression, cervical cancer, screening, disease mapping

## Abstract

**Background:**

Geographically weighted Poisson regression (GWPR) was applied to the relation between cervical cancer disease incidence rates in England and socio-economic deprivation, social status and family structure covariates. Local parameters were estimated which describe the spatial variation in the relations between incidence and socio-economic covariates.

**Results:**

A global (stationary) regression model revealed a significant correlation between cervical cancer incidence rates and social status. However, a local (non-stationary) GWPR model provided a better fit with less spatial correlation (positive autocorrelation) in the residuals. Moreover, the GWPR model was able to represent local variation in the relations between cervical cancer incidence and socio-economic covariates across space, whereas the global model represented only the overall (or average) relation for the whole of England. The global model could lead to misinterpretation of the relations between cervical cancer incidence and socio-economic covariates locally.

**Conclusions:**

Cervical cancer incidence was shown to have a non-stationary relationship with spatially varying covariates that are available through national datasets. As a result, it was shown that if low social status sectors of the population are to be targeted preferentially, this targeting should be done on a region-by-region basis such as to optimize health outcomes. While such a strategy may be difficult to implement in practice, the research does highlight the inequalities inherent in a uniform intervention approach.

## Background

Regression is a well known statistical tool for exploring the relationship between target and explanatory variables [1]. Different types of regression models are used widely in ecological and disease research, for example, global regression modelling, multi-level modelling and Bayesian modelling for small area studies [2]. For example, regression has been used to explore the relations between limiting long-term illness, ethnicity and income in London [3]. However, global regression models are stationary in the parameters and, thus, geographical variation in the relations is ignored. Geographically weighted regression (GWR) is a well established technique that relaxes the stationarity decision implicit in global models, thereby allowing parameters to vary spatially [4-6]. This amounts to a non-stationarity decision. GWR can, thus, be used to examine spatial variation in relations (i.e., in the parameters that define those relations) and reveal spatial patterns in parameters. Information on local spatial variation in parameters can lead to greater understanding of the relations between the target and explanatory variables.

Global regression models have an important role in disease studies [7]. However, in such studies, it is assumed that the relation between disease rate (or disease incidence) and explanatory variables is spatially constant, which may not be the case. The decision to ignore potential local spatial variation in parameters can lead to biased results which may in turn lead to poor guidance being provided to healthcare practitioners and the general population. Local spatial variation can be important and meaningful in disease analysis, pointing to the key local risk factors associated with disease incidence. Such information may have important implications for policy makers.

Geographical information systems (GIS) are commonly applied in disease studies [2,8,9]. GIS facilitate the handling of spatially referenced data and allow visualisation of spatial patterns in disease and identification of local hotspots. The geographical referencing of data that allows application of GIS also allows application of GWR. GWR is well developed for different statistical modelling frameworks (e.g., Gaussian and Poisson models). In the context of disease studies, *Gaussian *GWR has previously been applied to long-term limiting illness in the northeast of England, and the results showed regional variation in the regression parameters [10]. Geographically weighted Poisson regression (GWPR) can be applied to model disease counts and incidence rates (the focus of this paper, and a common focus in disease studies).

Many studies have shown that ill health issues are related to the surrounding socio-economic and socio-economic deprivation conditions [11-13]. For example, children in Bangladesh with a working mother have been found to have a higher chance of suffering from diarrhoea than those who have mothers who stay at home [14]. Other studies have shown that such relations may also vary between regions and that such variation should be taken into account [15] to provide more representative modelling and more accurate prediction. One reason postulated for the importance of local variation in such relations has been local variation in ability to access healthcare services [16]. Ill-health condition may also be related to human behaviour which may be a function of social background as well as educational level.

The Black report [17,18] suggested that higher income populations commonly made better use of health services, and there are significant social inequalities in using local health services in England [19]. Some research showed evidence of inequalities in health care access due to age distribution, sex structure, local deprivation conditions, and ethnic mix [16,19,20]. Such factors may explain variation in willingness to attend regular screening, and such factors may vary spatially. Therefore, socio-economic conditions and deprivation may be correlated with ill-health condition either directly, or through the effect of social conditions on poor service uptake [17].

Cancer is a common cause of death globally, with cervical cancer the second most common cancer for women worldwide [21,22]. The number of cases of cervical cancer is increasing, with about 471,000 new diagnostic cervical cancer cases per year worldwide [23]. About 80% of cervical cancer incidence cases occur in low income countries [22] while 70% of all cancer deaths in 2007 occurred in low and middle-income countries [24].

The National Statistics Report revealed differences in incidence in cervical cancer in the UK between manual and non-manual social classes, with a higher incidence in manual social classes [25]. In 2006 there were 2,873 new diagnostic cases and by 2007 there were 2,828 new diagnostic cases in the UK [23,26]. It is, thus, important to understand the risk factors associated with cervical cancer. Sexual behaviour is considered to be one of the main risk factors, as research has revealed an association between Human Papilloma Virus (HPV) and cervical cancer development [27]. In particular, HPV 16 and 18 are highly related to cervical cancer development [28-30]. It is estimated that 99% of cervical cancer cases are related to HPV infection [22]. Age is considered to be one of the risk factors associated with cervical cancer incidence, while other causal factors include family history, and female reproductive history. It is likely that cervical cancer development is also related to further associated factors.

Given the above evidence, it is important to understand the relations between cervical cancer disease risk and deprivation conditions, social status and family structure factors. Knowledge of such relations may be of use in planning screening programmes to reduce risk through early detection. In addition, such knowledge may be used to underpin resource allocation and service access design in relation to observed inequalities (e.g., screening programmes).

The aim of screening programmes is to detect abnormal or cancerous cells at an early stage because patients are expected to respond better to treatment at early disease stages. A screening programme can increase the chances of detecting cancerous and especially pre-cancerous cells at early disease stages so that the cancer incidence rate may be reduced and, thus, the likelihood of survival may be increased [21,23]. Early detection is a cost-effective and life saving strategy for chronic disease when the disease is still highly curable or preventable at early disease stages. The survival rate for cervical cancer in England and Wales between 1971 to 1999 was up to 80% for a one year period, 50-60% for a five year period and 40-50% for a 10 year period [31]. Importantly, the NHS Annual Screening Review Report [32] and the Cervical Screening Pocket Guide [23,33] suggested that the UK's cervical cancer screening programme can prevent about 75% of cervical cancer cases on average if all female patients attend screening regularly [34]. However, there has been concern that (i) the highest risk population is not tested sufficiently frequently and (ii) those with a positive test result are not followed-up and treated properly [33].

The aim of this research was to explore the spatial pattern in the relations between cervical cancer incidence and a set of socio-economic and deprivation conditions, social status and family structure factors in England using GWPR. The analysis has implications for the UK National Cervical Cancer Screening Programme.

## Methods

### Poisson regression

When modelling disease cases (count data) and particularly for rare diseases with low numbers of cases, the Poisson model is an appropriate regression model [35,36]. Many disease analysis studies over small areas have applied the Poisson model to describe the disease distribution [2,8,36].

In practice, the standardized mortality ratio (SMR) is commonly used to measure and compare regional mortality rates. In this research, the property of interest is the incidence rate rather than mortality rate, and so the standardized incidence rate (SIR) was used [13]. The SIR is defined as [13,37];

(1) $$SIR_{i}=\frac{y_{i}}{E_{i}}=\theta_{i},$$

Where *y_i _*is the number of observed incidence cases, and *E_i _*is the expected number of cases for region *i*, where *i *= 1, 2, ..., *N*. The expected number of cases *E_i _*is based on the overall incidence rate *r_g _*applied to the demographic structure [37]. The expected number of cases was calculated by using the normalized incidence rate *r_g _*per age group. This rate was normalized by multiplying by the ratio (~2.4) between total cervical cancer cases (the data used here) and new diagnosed cases (the data used in the Cancer Research UK rates). The normalized rate was then multiplied by the female population *p_gi _*within that age group in region *i*, where *g *is the age group. The female population per age group was determined from the 2001 UK Census of those aged between 25-29, 30-34, ..., 80-84 and is defined as;

(2) $$r_{g}=\frac{\underset{g}{\sum }y_{g}}{\underset{g}{\sum }p_{g}}$$

where, *g *is the age group

(3) $$E_{i}=\underset{g}{\sum }r_{g}p_{gi}$$

Since SIR is a standardized indicator of incidence rate, it varies around one; if the rate is above one, the observed incidence is greater than expected; if the rate is less than one, the observed incidence is less than expected. The Poisson regression model can be written as [4,6];

(4) yi~Poisson(Hi exp(f(xi))

The link between the target variable and *K *covariates can be described by a function *f*(*x_i_*). *H_i _*is the offset variable, which is a measurement unit of exposure for region *i*. Most disease studies based on the Poisson distribution framework use the expected number of cases *E_i _*as the offset variable.

### Geographically Weighted Poisson Regression (GWPR)

GWR is a well established technique that can be used to examine spatial variation in relations (i.e., non-stationary regression parameters). Information on local variation in parameters can lead to greater understanding of the relations between the target and explanatory variables. When GWR was first developed, the Gaussian model was used in disease studies [10]. This section expands on GWR to describe the GWPR method. The theory and materials in this section are covered by Fotheringham *et al. *[4] and Nakaya *et al. *[6] and so only a summary is provided. The traditional linear regression model is generally defined as below in equation (5);

(5) yi=Ei exp(β0+ ∑kβkxki+εi)logyi= logEi+(β0+ ∑kβkxki+εi)

Where *β*_0 _is the intercept, the *β_k _*are the coefficients of the covariates *k *and *ε_i _*is the error term for region *i *= 1, ..., *N*. The estimated parameters are constant over space. GWR is an extension of the above traditional model in which all parameters are allowed to vary over space. The model framework is defined as below [4,6];

(6) $$\begin{array}{l} y_{i}=E_{i}\exp(\beta_{0}(u_{i},v_{i})+\sum_{k}\beta_{k}(u_{i},v_{i})x_{ki}+\varepsilon_{i})\\ \log y_{i}=\log E{+}_{i}(\beta_{0}(u_{i},v_{i})\\ \;\;\;\;+\sum_{k}\beta_{k}(u_{i},v_{i})x_{ki}+\varepsilon_{i}) \end{array}$$

where, (*u_i_*, *v_i_*) is the coordinate of the *i*th region, which describes the location of *i*. For polygons, such coordinates are normally defined as the centroid of region *i *recorded as a two-dimensional vector. *β*_0 _(*u_i_*, *v_i_*) is the intercept for location *i*, *β_k _*(*u_i_*, *v_i_*) is a realisation of the continuous function of *β_k _*at region *i *and *ε_i _*represents the error term and it is assumed to follow a Gaussian distribution with mean zero and variance *σ *^2 ^.

As discussed at the beginning of this section, the Poisson model is generally more appropriate for disease data. The GWPR model can be written as [4,6];

(7) yi~Poisson(Ei exp(∑kβk(ui,vi)xki))

Such a model allows the parameters to vary geographically [6]. The model can be calibrated based on a kernel regression method, which allows users to estimate the geographical variation in model parameters with a given spatially weighted kernel. The optimal size of the kernel is usually estimated through calibration.

To estimate the GWPR parameters, a local likelihood methodology was applied to estimate the local parameters at location *i *by maximizing the geographically weighted log-likelihood function in equation (8) [4,6,38]:

(8) $$\begin{array}{l} \max L(u_{i},v_{i})=\sum (-{\hat{y}}_{j}(\beta (u_{i},v_{i}))\\ +y_{j}\log{\hat{y}}_{j}(\beta (u_{i},v_{i})))w_{ij}(\left\|\left(u_{i},v_{i})-(u_{j},v_{j}\right)\right\|) \end{array}$$

where, *w_ij _*is the weighted value and (*u_i_*, *v_i_*) - (*u_j_*, *v_j_*) represents the distance between regression point *i *and data point *j*.

The weighting function is defined by the kernel type and the size of kernel (referred to here as the bandwidth). The weighting function *w_ij _*determines the geographical weight of the *j*th observation at the *i*th regression point. In theory, the weight should decrease gradually as the distance between *i *and *j *increases, eventually, converging to or reaching zero. Parameter estimates are highly related to kernel size such that choice of kernel is an important consideration. There are two commonly employed types of kernel: (i) the Gaussian kernel and (ii) the bi-square kernel:

(i) Gaussian kernel with fixed bandwidth in which each local regression model has the same spatial size of kernel, but each kernel may cover a different number of data points. The function is defined as [4];

(9) $$w_{ij}=\exp\left(-\frac{1}{2}\frac{d_{ij}}{d}\right)$$

where *d_ij _*is the distance between regression point *i *and data point *j *and *d *is a non-linear parameter (bandwidth). The closer a data point *j *to regression point *i*, the larger the weight given [4].

(ii) Adaptive method with bi-square kernel, in which the bandwidth covers the same number of data points with non-zero weight within each regression model. Any points outside the bandwidth *d *have zero weight and are excluded from the local regression. This adaptive kernel is a common choice, especially, when the sampling density varies greatly across space. The function is given as [4];

(10) $$w_{ij}=\left\{\begin{array}{cc} {\left[1-{\left(\frac{d_{ij}}{d}\right)}^{2}\right]}^{2} & d_{ij}<d\\ 0 & otherwise \end{array}\right)$$

The choice of bandwidth has an important role in relation to the level of smoothing of the outputs. A larger bandwidth generally produces greater smoothing. An optimal bandwidth may be selected in terms of some criterion. In practice there are three common means of choosing the bandwidth, (i) subjective, (ii) smallest cross-validation error and (iii) smallest Akaike information criterion (AIC). In this paper, parameter estimates were calibrated in a point-wise way, and the kernel size with minimum adjusted Akaike Information Criterion (AICc) was selected as optimal (and the Bayesian Information Criterion (BIC) was also considered).

### Geographically weighted Poisson regression statistics

In GWPR, all parameter estimates are made using an iterative procedure that continues until convergence; once the prediction at location *i *has changed, the prediction at *j *may also be affected if *j *is within the bandwidth of the regression point *i*. Therefore, it is necessary to maximise equation (8). The method for solving this equation is to apply a type of local Fisher scoring procedure, which is called iteratively reweighted least squares [6]. All the methods in this section are covered by [4,6] and only a brief summary is given.

The estimation of local parameters is given in equation (11),

(11) $$\begin{array}{c} \beta^{\left(l+1\right)}\left(u_{i},v_{i}\right)={\left(X^{t}W\left(u_{i},v_{i}\right)A{\left(u_{i},v_{i}\right)}^{\left(l\right)}X\right)}^{-1}\\ \;\;\;\;\;\;\;\;\;X^{t}W\left(u_{i},v_{i}\right)A{\left(u_{i},v_{i}\right)}^{\left(l\right)}z{\left(u_{i},v_{i}\right)}^{\left(l\right)} \end{array}$$

where, *z*(*u_i_*, *v_i_*)^(*l*) ^is a vector of adjusted dependent variables, *A*(*u_i_*, *v_i _*)^(*l*) ^is the variance weights matrix associated with Fisher scoring for each location *i*, *W *(*u_i_*, *v_i _*) is the diagonal spatial weights matrix for location *i*, *X *is the design matrix, *X ^t ^*is the transpose of *X*, and *l *represents the number of iterations. Finally, the parameters are estimated for each location *i*, until the estimates converge.

### Standard error

Since the aim is to estimate local parameters, it is important to calculate the local standard errors. Such standard errors take account of variation in the data, which can be used to compare the estimates. If there are only a few points within the regression bandwidth area or those regression points are far away from the regression point the local error may be large. The local standard errors are highly related to the *j *points (data), which lie within the regression bandwidth. So the locations of the regression point *i *and data points *j *determine the standard error of the parameters.

In this section, estimation of the local standard errors is considered. The local parameter estimates are defined as in equation (11). When the estimation process has converged the number of iterations *l *can be ignored and the equation redefined as [4,6];

(12) $$\begin{array}{c} \hat{\beta }\left(u_{i},v_{i}\right)=C\left(u_{i},v_{i}\right)z\left(u_{i},v_{i}\right)\\ \;\;\;\;\;\;\;\;\;={\left(X^{t}W\left(u_{i},v_{i}\right)A\left(u_{i},v_{i}\right)X\right)}^{-1}\\ \;\;\;\;\;\;\;\;\;X^{t}W\left(u_{i},v_{i}\right)A\left(u_{i},v_{i}\right)z\left(u_{i},v_{i}\right) \end{array}$$

where, *A*(*u_i_*, *v_i_*) and *z*(*u_i_*, *v_i_*) are calculated based on the converged estimates of $\hat{\beta }\left(u_{i},v_{i}\right)$. The *z*(*u_i_*, *v_i_*), are assumed to follow a normal distribution with zero mean and variance-covariance *A*(*u_i_*, *v_i_*)^-1^. The asymptomatic variance-covariance of the *k*th parameter estimate is given by,

(13) $$\text{cov}\left(\hat{\beta }\left(u_{i},v_{i}\right)\right)=C\left(u_{i},v_{i}\right)A{\left(u_{i},v_{i}\right)}^{-1}C{\left(u_{i},v_{i}\right)}^{t}$$

where, the standard error of the *k*th parameter estimation is given by,

(14) $$Se\left(\beta_{k}\left(u_{i},v_{i}\right)\right)=\sqrt{\text{cov}{\left(\hat{\beta }\left(u_{i},v_{i}\right)\right)}_{k}}$$

### Model measurement and comparison for GWPR

The coefficients vary continuously over space. Therefore, it is almost impossible to achieve universally accurate estimation. Models with very few data points lead to large standard errors in local parameter estimation. On the other hand, a model with a large number of data points can provide more reliable local parameter estimation. However, such models may contain a large amount of bias as the distances between regression point *i *and data points *j *increase. Thus, it is important to obtain a balance between the bias and variance of the parameters being estimated.

An optimal size of bandwidth is needed to provide unbiased estimation of the local parameters. There are many indicators available, such as the Akaike information criterion (AIC) and Bayesian information criterion (BIC). For GWR, it is common to use the AIC to assess the performance of the fitted model with certain covariates and for a given bandwidth. One way of achieving the right balance is to use some model selection indicators. There are many available indicators. In this study, the adjusted AIC was used to assess the performance of the bandwidth size and BIC was used as an alternative measurement. AIC was developed by Akaike in 1974 [39,40] to measure the performance of statistical models. The AIC of the model with bandwidth *d *is given as [4,6];

(15) AIC(d) = D(d) + 2K(d)

The deviance is represented by *D *and the effective number of parameters is represented by *K *with bandwidth *d*. The model with the smallest AIC (i.e., the model with optimal bandwidth) is called the minimum AIC estimator (MAICE). In practice, if the difference in AIC between two models is less than or equal to two, there is no significant difference between the two models.

In some situations the AIC can perform poorly or may even be biased, for example, when there are too many parameters with a small number of observations [40,41]. To avoid such biased estimation from AIC, Sugiura [41] derived a second order variant of AIC which is called the c-AIC, and Hurvich and Tsai [42] incorporated a small sample (second order) bias adjustment which led to a criterion called AICc.

(16) $$\begin{array}{lll} AICc\left(d\right) & =D\left(d\right)+2K\left(d\right)+2\frac{K\left(d\right)\left(K\left(d\right)-1\right)}{N-K\left(d\right)-1}\; & \text{(1)}\\ & =AIC\left(d\right)+2\frac{K\left(d\right)\left(K\left(d\right)+1\right)}{N-K\left(d\right)-1}\; & \text{(2)}\\ & \; & \text{(3)} \end{array}$$

The other bandwidth selection criterion that can be used in GWPR is called the BIC [4], the calculation of which is given by,

(17) $$BIC=-2\log\left(L\right)+K\log_{e}\left(N\right)$$

where, *L *is denoted as the model likelihood, *K *is the effective number of parameters and *N *is the total number of regions. BIC was derived from Bayesian theory, where each of a discrete number of candidate models have equal prior probabilities (the prior distributions on the model parameters). The model with the smallest BIC is the better fitted model compared to the other candidate models. AICc was used here to compare candidate models, and BIC was used as an alternative.

### Data collection

Any locations with a small number of incidence cases and deaths per district or unitary authority (i.e. 0-5) were represented as missing data (NAs) for reasons of confidentiality. In the modelling, such NAs were treated as truncated data. Further details of the data collection are described below.

### Cervical cancer and socio-economic data

Two sets of data were included for analysis; cervical cancer count data for 2004 and explanatory variables for 2001. The cervical cancer count data were provided by the Association of Public Health Observatories (APHO), which represents the set of nine Public Health Observatories (PHO) in England (Table 1). In total, 7179 cervical cancer cases and 2391 deaths were recorded in 2004. The data are represented at the district and unitary authority levels of the Cancer Registries in England. At the beginning of this study the data were subjected to a Chi-square goodness of fit test, which showed that the data approximately follow a Poisson distribution.

**Table 1:** The set of nine Public Health Observatiories (PHO) in England.

Public Health Observatory (PHO)	Number of regions in PHO
1. South West	45

2. South of England	67

3. London	33

4. East of England	48

5. East Midlands	40

6. West Midlands	21

7. North West	23

8. Yorkshire and Humber	43

9. North East	34

**Figure 1:**
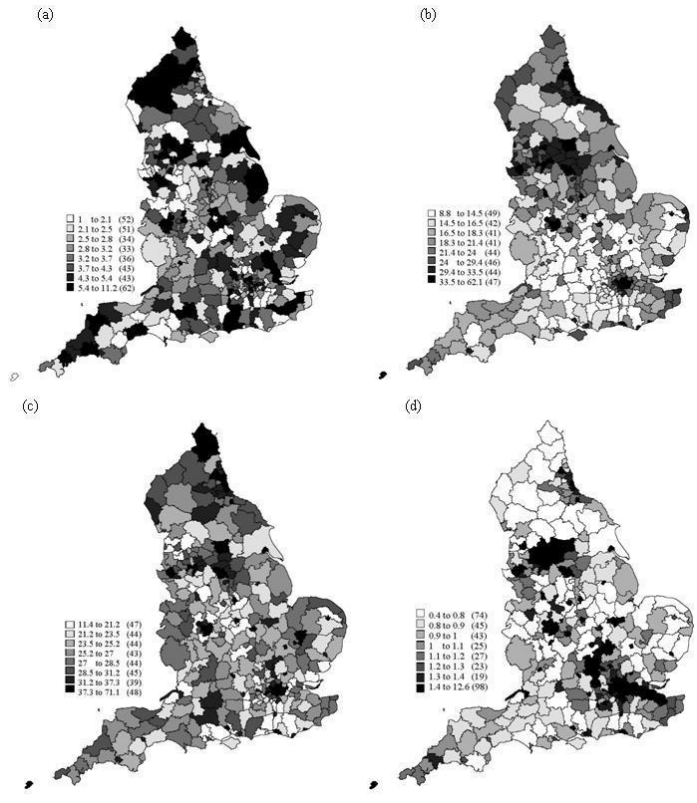
**The spatial distribution of the components of the Townsend index, which are shown for reference: (a) percentage unemployed, (b) percentage of households with no car, (c) percentage of households not owned and (d) percentage of rooms occupied by more then one person**.

In this research, the Townsend index was chosen to measure deprivation. It is a common index and it has been used widely in health studies. The Townsend index comprises four scores (Figure 1) that represent socioeconomic deprivation (Table 2).

**Table 2:** Summary of explanatory variables used as indicators in the regression analysis.

Property	Covariate	Description	Table from UK census 2001
**Deprivation**	(i) Townsend index score	Includes: (i) unemployment,	KS009a Economic activity: all persons (from the key statistics)
		(ii) households not owned,	KS018 Tenure (from the key statistics)
		(iii) car ownership (all households with no cars or vans) and	KS017 Cars or vans: all households (from the key statistics)
		(iv) over-crowded housing (over one person per bedroom)	UV 058 Person per room (from the census area statistics univariate tables)

**Family structure**	(ii) Female marital status: proportion of single females	Defined as single (never married) + divorced + widowed	ST002 Age by sex and marital status
	(iii) Female marital status: proportion of married females	Defined as married + remarried + separated (but still legally married)	
	(iv) Households with lone parents: all	All lone parents (both male and female)	KS022 Lone parents households with dependent children
	(v) Households with lone parents: females only	Female lone parents only	

**Social grade (proportion)**	(vi) Proportion of social grade IV + V	Includes:	UV050 Approximated social grade IV and V (low socio-grade)
		(i) Grade IV: semi- skilled and unskilled manual workers	
		(ii) Grade V: on state benefit, unemployed, lowest grade workers	

The calculation of the Townsend score for each variable is defined below. Let *V_ih _*be the value of each socio-economic variable, for variables *h *= 1 to 4 and *i *= 1 to *N *areal units in the data. The Townsend score *z_ih _*is a standardized measure for each of the four deprivation variables obtained by subtracting from *V_ih _*the mean *m_ih _*and dividing by the standard deviation *σ_ih _*as below.

(18) $$z_{ih}=\frac{V_{ih}-m_{ih}}{\sigma_{ih}}\;\text{where }i=1,2,\;\ldots ,\;N\;\text{and }h=1,2,3,4$$

Both variables (i) unemployed population and (iv) over-crowded housing were transformed by a natural *log q *= ln(*s *+ 1), where *q *is the value after the transformation and *s *is the observed value of the socio-economic variables, to make the variables approximately normally distributed.

The Townsend index is calculated from the sum of *z_ih _*as follows:

(19) $$Z_{i}=\overset{4}{\underset{h=1}{\sum }}z_{ih}\;\text{where }i=1,2,\;\ldots ,\;N\;\text{and }h=1,2,3,4$$

The greater the *Z *value the greater the deprivation.

**Figure 2:**
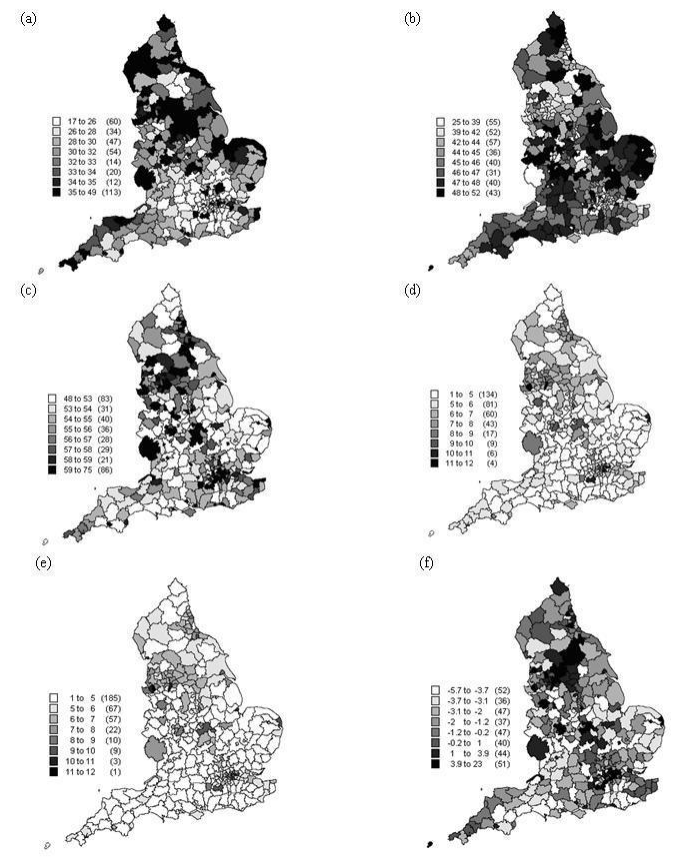
**The spatial distribution of the covariates used in the analysis (a) percentage of socio-grade IV and V, (b) percentage of married female population, (c) percentage of single female population, (d) percentage of lone parent households, (e) percentage of female lone parent households and (f) the Townsend index**.

Other variables were added to represent family structure and social status. Thus, the set of explanatory variables comprises the Townsend Index plus family structure (the proportion of married females, proportion of single females, proportion of lone parents including male and female parents, and proportion of female lone parents) and low social status. All explanatory variables are listed in Table 2 and mapped in Figure 2. All data were downloaded from the UK Census of 2001. Since the census is carried out once every ten years the closest matched year to 2004 was 2001.

### Truncated data

Because of confidentiality restrictions, data were aggregated as summary counts for regions rather than being provided for individuals. For districts with less than or equal to five cases, the number of incidence cases was not disclosed in order to protect the patients' privacy. Closed, missing or truncated data are common in disease studies. While it is possible to exclude or remove truncated data from analysis such an approach would amount to information being discarded, potentially reducing predictive power [43]. Therefore, within the analysis all the counts between 0-5 were treated as truncated data. In total, these truncations were applied to 21 regions (6%).

One way of dealing with truncation is to estimate the basic true mean of the Poisson distribution from the data, including the missing data. Accepting the estimated means to be true, then a random number can be drawn from the Poisson distribution for each of the regions. For each region, a stream of random numbers was drawn and the first 100 random numbers between 0-5 were used to replace the missing data. 100 sets of missing values were produced, and the GWPR models were fitted 100 times, each time with a different set of missing values. Then the mean and variance of the predictions was calculated.

Since there were 100 different sets of realisations for the missing data, the GWPR model was fitted 100 times and the average of the predictions for the 100 models was estimated:

(20) $$E\left({ŷ}_{i}\right)=\frac{\overset{100}{\underset{n=1}{\sum }}{ŷ}_{in}}{n}$$

where, *n *is the number of GWPR models, ${ŷ}_{in}$ is the prediction for the *n*th model, and $E\left({ŷ}_{i}\right)$ is the expectation of ${ŷ}_{i}$, the overall average prediction from the 100 GWPR model predictions.

The variance for the 100 predictions was estimated to characterise the overall variation resulting as a function of the uncertainty due to truncating the distribution. The variance provides information on prediction uncertainty and parameter estimation uncertainty. The variance was calculated as;

(21) $$\text{var}\left({ŷ}_{i}\right)=\frac{\overset{n}{\underset{i=1}{\sum }}{\left({ŷ}_{in}-E\left({ŷ}_{i}\right)\right)}^{2}}{n-1}$$

where, $\text{var}\left({ŷ}_{i}\right)$ is the overall variance between the *n *= 100 models.

## Results

### Global model

To examine the possible determinants of the geographical patterns in cervical cancer incidence, a traditional global Poisson regression model was fitted with an offset equal to the expected number of cases based on the demographic composition of each region. All covariates were significant to the observed incidence. For full details of measurements of all other candidate models please refer to Table 3. The final fitted global Poisson regression model is defined as:

**Table 3:** Summary statistics of model comparisons.

Model	Variables	Kernel	AICc (global)	BIC (global)	AICc (local)	BIC (local)
1	Townsend index score	91	853.02	860.73	640.38	709.92

2	Female single proportion	91	849.38	857.09	651.32	725.37

3	Female married proportion	91	849.38	857.09	651.32	725.37

4	All lone parents proportion	91	750.37	858.08	594.87	666.85

5	Female lone parents proportion	91	754.68	762.32	597.28	669.28

6	G4 + G5 proportion	91	612.97	620.67	539.32	610.35

7	G4 + G5 + Female lone parents proportion	91	614.88	626.41	539.80	642.00

8	G4 + G5 + Townsend index score	91	612.22	623.76	538.49	637.54

9	G4 + G5 + Female married proportion	91	613.47	625.01	539.67	642.53

10	G4 + G5 + All lone parents proportion	91	614.96	626.50	539.21	641.45

G4 and G5 represent the proportion of low social grade (IV+V) population in region *i*. G4 (Grade IV) represents the proportion of semi-skilled and unskilled manual workers. G5 (Grade V) represents the proportion of the population on state benefit, the unemployed, and the lowest grade workers

(22) ŷi=Ei exp(-0.718+2.832xG45i)

where, *x_G_*_45_*_i _*represents the proportion of low social grade population (i.e., from social status IV and V) (Table 2). *x_G_*_45_*_i _*was found to be significant and associated to cervical cancer incidence rate at the global level. The AICc of this model is 612.97 and BIC is 620.67 (Model 6 in Table 3), which can be used to compare this model with other models. The proportion of low social status population includes semi-skilled manual workers, unskilled manual workers, people on state benefit, the unemployed and the lowest grade workers (Table 2). When this proportion of the population increases the incidence rate is likely to increase. The ratio between the likelihood of cervical cancer for the low social grade population and that for the high and medium social grade population is about 2.8. The *x_G_*_45_*_i _*variable may reflect the amount of general knowledge about personal ill-health issues or the ability or willingness to access local healthcare services including attending regularly the National Cervical Cancer Screening Programme.

### GWPR analysis

The global regression model showed that the proportion of low social status population is significant covariate of incidence rate at the global level, but such a model may mask potential local spatial variation in the relation between incidence rate and the covariates. Thus, the GWPR model was applied.

**Figure 3:**
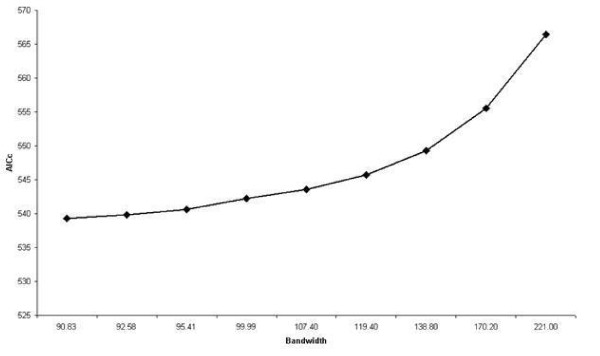
**The AICc plotted against kernel bandwidth**.

As summarized above, the use of an adaptive weighting function and the optimal bandwidth were selected based on the smallest AICc in Table 3. Figure 3 shows the AICc plotted against kernel size, and that the optimal bandwidth is 91 regions. The size is relatively large, which may be due to the sample size being small in most of the regions. Therefore, a large bandwidth was required to cover sufficient data to predict reliably.

As described in the methods section, the models were compared using the AICc; the smallest AICc values were assumed to provide the best fitting model from the candidate models. One of the datasets from the set of 100 randomly imputed datasets is shown in Table 3. From Table 3, it can be concluded that the best fitting model is the GWPR model with the proportion of low social status population as a covariate (model 6 in Table 3). The final fitted model is given as (model 6 in Table 3);

(23) $$\log{ŷ}_{i}\left(u_{i},v_{i}\right)=\log E_{i}+\left(\beta_{0}\left(u_{i},v_{i}\right)+\beta_{1}\left(u_{i},v_{i}\right)x_{G45}\left(u_{i},v_{i}\right)\right)$$

**Figure 4:**
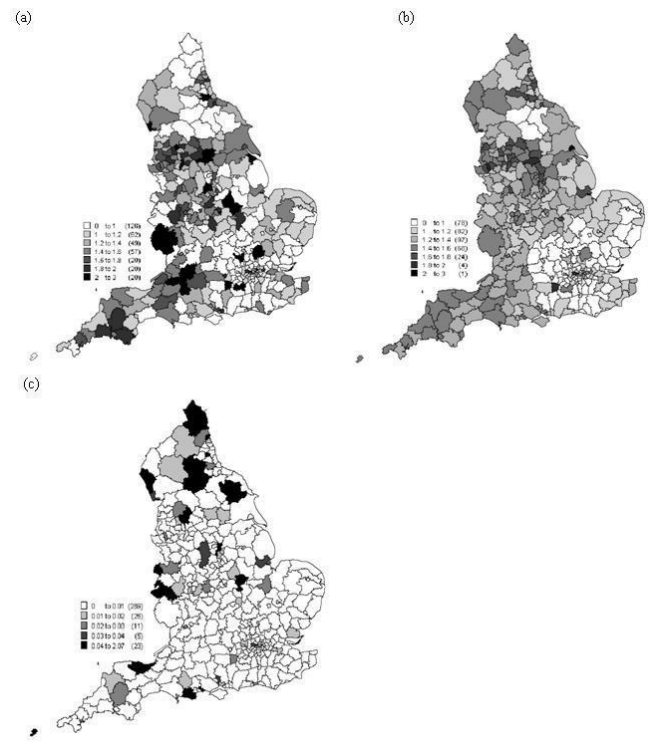
**The spatial distribution of (a) the raw SIR, (b) the mean of the predictions of SIR from the 100 local models, (c) the variance of the predictions of SIR from the 100 local models from model 6 in Table 3**.

**Figure 5:**
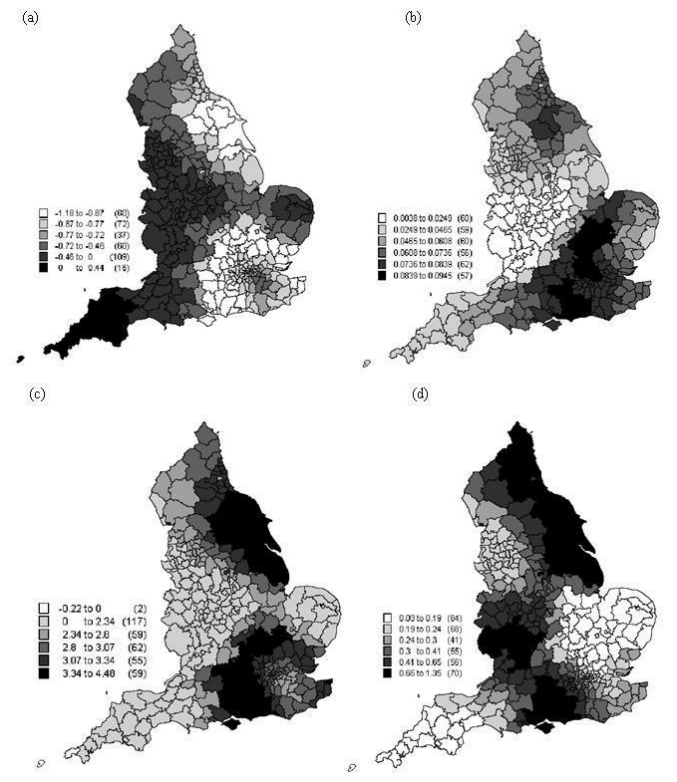
The spatial distribution of estimated parameters: (a) mean of *β*_0*i*_, (b) variance of *β*_0*i*_, (c) mean of *β*_1*i *_and (d) variance of *β*_1*i*_

The overall means and variances of the predictions of SIR from the 100 local models are displayed in Figure 4b and 4c). The average estimated means and variances of ${\hat{\beta }}_{0}\left(u_{i},v_{i}\right)$ and ${\hat{\beta }}_{1}\left(u_{i},v_{i}\right)$ from the 100 samples are displayed in Figure 5.

**Figure 6:**
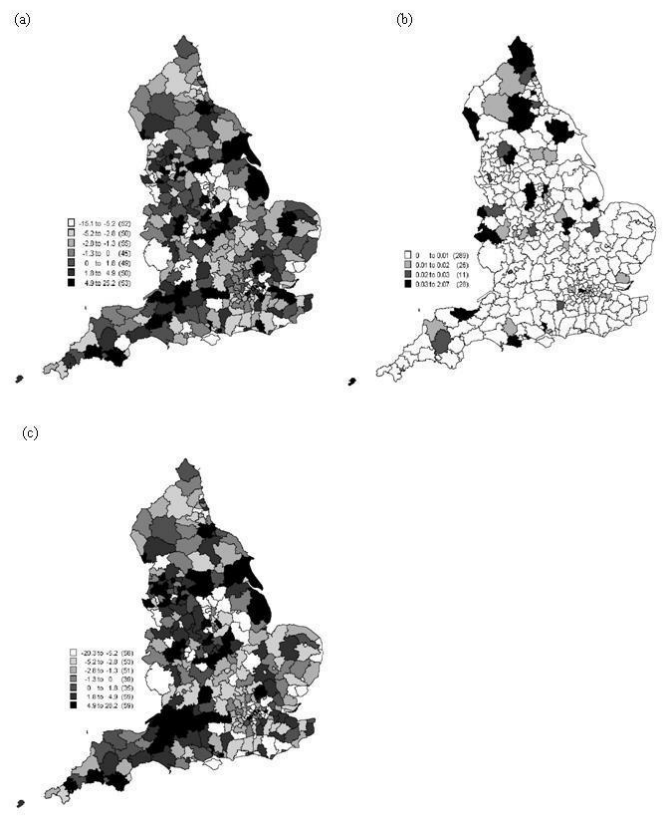
**The spatial distribution of (a) the mean of the residual values from the 100 local models from model 6 in Table 3, (b) the variance of the residual values from the 100 local models from model 6 in Table 3, (c) the residual values from the global model (for comparison)**.

**Figure 7:**
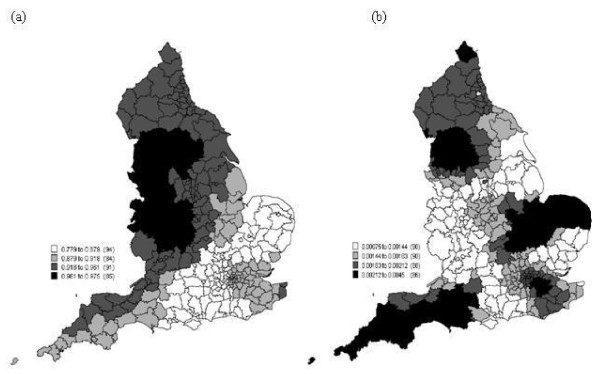
**The spatial distribution of (a) the mean of the *R*^2 ^from the 100 local models and (b) the variance of the *R*^2 ^from the 100 local models from model 6 in Table 3**.

The GWPR analysis revealed an interesting positive *local *relationship between incidence rate and the proportion of low social status population, which was hidden from the global model. The raw data (Figure 4a) and the mean of the 100 predictions (Figure 4b) have a similar spatial pattern and the variance in Figure 4c reveals only a small amount of variation between the 100 models, meaning that data truncation had little effect on the results. The map of the mean of the 100 maps of residuals from the 100 local models (Figure 6a) exhibits little spatial correlation and the variance of these residuals (Figure 6b) again reveals only a small amount of variation between the 100 models. The *R*^2 ^values of the local models in Figure 7a are generally large, between 0.78 to 0.98, and the variance of the *R*^2 ^values from the 100 models is relatively small between 0.00075 to 0.0045 (Figure 7b).

The correlation is generally positive, as for the global model, but the effect of and contribution from the low social status variable (i.e., the estimated coefficient) varies between 0.07 and 4.40 times across England. There is a greater contribution from this variable in the south and north-east of England (see Figure 5c). Low social status population had far less effect in the west of England. This might be related to population structure, due to the higher proportion of elderly population in the west of England compared to the national average. Two regions exhibit a negative relation (-0.22 and -0.04 times) which are Penwith (South West England) and Scilly. From (Figure 5a and Figure 5c), it is clear that the contribution from low social status varies over space, and when *β*_0*i *_decreases then *β*_1*i *_increases. The intercept *β*_0*i *_varies between -1.18 and 0.44.

### Stationary parameters

It is interesting to examine which explanatory variables are fitted adequately using a model with stationary parameters, and which variables required a non-stationary model. The method used to answer this question is to compare the inter-quartile range at the local level and the standard error at the global level. If the local inter-quartile range is twice the global standard error, then the variable requires a non-stationary model to represent it adequately [4] (Table 4).

**Table 4:** Simple test for non-stationarity.

Parameters	2* S.E. (Global)	Inter-quartile range (Local)	Stationary or non-stationary variable
**Intercept**	0.06	0.46	Non-stationary

**Proportion of lower social grade (G4 + G5) population**	0.18	1.05	Non-stationary

Table 4 shows that both *β*_0*i *_(intercept) and *β*_1*i *_(low social grade population G4 and G5) have an inter-quartile range more than twice the global standard error. This indicates that the socio-economic variables (i.e. low social grade population *G4 *and *G5*) are better fitted by a non-stationary model in GWPR than using a global regression. A non-stationary model allows greater prediction power, and as a result, leads to greater understanding of the relation between incidence rate and the proportion of low social grade population and how it varies over space in relation to deprivation conditions regionally.

## Discussion

From the above results it is clear that the relation between incidence rate and proportion of low social status population varies spatially. Specifically, the estimated parameters mapped in (Figure 5a and Figure 5c) vary spatially. Low social status was the most significant factor related to cervical cancer incidence rate. The local coefficient ${\hat{\beta }}_{1i}$ (Figure 5c) showed how the proportion of low social status of population contributed to the incidence rate. The coefficient mapped in Figure 5c revealed different contributions across England of between 0.07 and 4.40 times. A larger contribution is evident in the south and north-west of England than in the west of England. This suggests that the proportion of low social status in south and north-west England has a greater effect than in the Midlands and south-west of England. Therefore, global models are not suitable to describe the relationships between cervical cancer risk and explanatory variables. South-west England (e.g. Cornwall) has a lower incidence rate and also a small estimated coefficient. In particular, Penwith and Scilly had a negative relation with incidence rate. This might have arisen due to the population structure; the proportion of elderly people is larger in the west of England than the south of England. It might also arise because the locations are relatively isolated, which hinders prediction due to lack of local data points.

In terms of prediction, some regions were under-predicted while others were over-predicted. There are two important cases (i) those regions that are relatively large (i.e. the size of the cell polygon is large), and (ii) those regions that include extreme cases. For the first case, when the regions are relatively large, the distance between the data point *i *and regression point *j *is large, so that the accuracy of the prediction may be reduced. In the second case, the prediction can be biased because of the influence of extreme neighbours.

In Figure 6(c), the residual values from the global model seem to exhibit a small amount of autocorrelation. This autocorrelation was measured using Moran's *I *index as 0.04 with a *z*-score of 6.60 from Table 5. Moran's *I *index was also calculated based on restricting the local distance to 100 km, in which case Moran's *I *was 0.08, with a *z*-score equal to 6.78. These results suggest that the global model based on proportion of low social status cannot explain the spatially correlated variation in incidence rate. Thus, some potentially explanatory variables may be missing from the model (e.g., sexual behaviour, personal HPV history, family history etc.). The residual values from the local model (Figure 6a) exhibit a random pattern with a Moran's *I *index of 0.0012 and *z*-score 0.63 (Table 5). Moran's *I *was also calculated based on restricting the local distance to 100 km, in which case Moran's *I *was - 0.0021 with a *z*-score equal to 0.012. Thus, the GWPR model removed the problem of autocorrelation in the residuals.

**Table 5:** Moran's *I *results for global and local models.

	Global model (whole area)	Global model (local distance restricted to 100 km)	Local model (whole area)	Local model (local distance restricted to 100 km)
**Moran's Index**	0.04	0.08	0.0012	-0.0027

**Expected Index**	-0.0028	-0.0028	-0.0028	-0.0028

**Variance**	0.000040	0.00014	0.000040	0.00014

***z*-score**	6.60	6.78	0.63	0.01

A disadvantage of using the set of socioeconomic covariates used in the Townsend index is that they account for socio-economic conditions, but no family structure information is included. Thus, in this research information was added on family structure. However, it is not possible to distinguish between individuals who are not able to buy a car and those who do not need a car. Those people who live in a main city (e.g. London) may not need a car since public transportation is more convenient. Similarly, it is not possible to distinguish between individuals who are not able to buy a property and those who do not want to buy a property. For example, it is more common for people who live in a main city to rent a flat than buy a house.

Most deprivation indices used commonly in the UK are obtained from the UK census. However, there are limitations in the use of census data, most notably that the data are aggregated into areal units (i.e. data are not available at the individual level) and some information is not available (e.g. personal income, environmental conditions). The analysis presented in this paper is, therefore, valid at the census unit scale only. Since deprivation indices are commonly represented on areal units and the units are likely to vary over space, some units may be relatively larger than others. For this reason the covariates may be sensitive to the size of denominator.

The Townsend variables used here measure local welfare and local socio-economic behaviour which can be useful in health care studies. These variables are also adopted in other deprivation indices (e.g. Carstairs and DoE81) [44]. Alternative indices (e.g. the multiple deprivation index) [45] could be applied to explore the impact on cervical cancer incidence of income, employment, education deprivation and living environment deprivation. However, since some variables (i.e. income and living environment deprivation) were not available at the national level for the study period, such analysis is beyond the present scope and will be considered in further work.

In the present research, a simple method was applied to deal with truncated data. Random numbers were drawn from a Poisson distribution to replace the missing data. However, Figures 4(c) and (Figure 5b and Figures 5d) show very limited variance around the predictions for those areas with missing data, which suggests that the results are not affected greatly by truncation. Further research is needed to explore other possible methods to solve the truncation problem. For example, [46] demonstrated an approximate Bayesian bootstrap method which would be interesting to apply here.

It is possible to go beyond determining which variables can be described adequately by a stationary process and which are best fitted by non-stationary models by applying a mixed GWPR. A mixed GWPR is a semi-parametric GWPR model; it allows some variables to vary spatially and others to remain constant in a single approach. This will be explored in future research.

Many studies [3,9,20,47] have suggested that poor health outcomes often appear in the most deprived areas. Some studies have demonstrated health care inequality in terms of patient needs and access to NHS services in England [16,20,30]. The relation between health outcomes and social status should be a concern to all governments that espouse ideals of equality. This research demonstrated a locally varying relation between cervical cancer incidence and low social status. This relation may be associated to some patient factors including (i) personal understanding of the cervical cancer programme, (ii) misunderstanding of the current screening policy with regard to age criteria between groups and (iii) lack of knowledge about preventing cervical cancer at early disease stages. However, further analysis is required to explore the underlying causes.

The GWPR results may be useful for policy makers engaged with reviewing current policy and services. For example, it is possible to target patients in at least two ways: (i) divide the population into risk groups according to their age and social status (e.g. low, medium and high risk), or (ii) divide the study area (England) into several regions with similar social status. Each of the groups might then be allocated a different screening programme (e.g. a different screening test or screening interval). From the financial viewpoint such a strategy may save resources or make better use of available limited resources. From the patient's point of view the benefit may be an increase in the chances of detecting and preventing long-term disease. In practice, it is unlikely to be practical to divide the population into risk groups or divide England into several regions with varying risk levels. However, the analysis does provide evidence for the inequality of cervical cancer screening at the local level.

An NHS study showed that the number of cases with Cervical Intra-epithelial Neoplasia (CIN3) has increased for women aged between 20 and 24 because of trends in sexual behaviour, with increasing numbers of young people becoming more active sexually when they are still in their mid-teens [48]. A recent study discussed the poor use of cervical cancer screening resources within current NHS practice [49]. This change in sexual behaviour arises partly because of socio-economic changes through time and from place to place. If that is true, then recognizing the associated risk factors may be useful for developing a long-term prevention strategy for cervical cancer. For example, it may be possible to improve sex education in local schools, teaching mid-teen pupils about protective sex and sexually transmitted infectious diseases.

A HPV vaccine is available, and some clinical studies in Italy and Germany showed that use of the vaccine significantly reduces the incidence of cervical cancer. Thus, the vaccine might be considered as a means of achieving increased efficacy and cost-effectiveness in screening programmes in the future [50,51], particularly for younger age groups.

For interventions such as the national screening programme, sex education in schools and for vaccination, it may be considered desirable to target preferentially the low social status sectors of the population (the global model). The results of this paper show that if such targeting were to be considered then it should be done on a region-by-region basis (the GWPR model).

## Conclusions

Traditionally, global regression models have been used to explore the relations between health outcomes and explanatory variables. However, such techniques do not account for spatial variation in the relations. This research demonstrated the use of GWPR to examine the relations between cervical cancer incidence rates and socioeconomic covariates across England. Cervical cancer incidence rates were found to vary spatially across England (e.g. Cornwall and the North of England had low incidence rates compared to the rest of England). Moreover, cervical cancer incidence was found to be associated with low social status and, importantly, this relation was found to vary spatially across England.

Spatial variation in the relations between incidence and socio-economic covariates means that in some places socio-economic status has a greater effect on incidence than in other places. This may reflect differences in personal behaviour, local differences in educational levels across the social classes, or differences in screening up-take rates over space. Ignoring such spatial variation could lead to inefficient resource usage nationally. To maximise the benefits of the national cervical cancer screening programme this research suggests that the low socio-economic status sectors of the population should be targeted, and in some places more so than in others.

## Competing interests

The authors declare that they have no competing interests.

## Authors' contributions

PMA conceived the original idea. PMA and MYEC designed the study, including the choice of methods. MYEC undertook the statistical analysis and drafted an initial manuscript. PMA, AKS and MYEC contributed to writing the final manuscript. All authors read and approved the final manuscript.
